# Neuropsychological and real-life functioning of adults with ADHD

**DOI:** 10.1007/s00702-021-02357-5

**Published:** 2021-06-12

**Authors:** Oliver Tucha, Anselm B. M. Fuermaier

**Affiliations:** 1 Department of Psychiatry and Psychotherapy, University Medical Center Rostock, 18147 Rostock, Germany; 2 Department of Psychology, Maynooth University, National University of Ireland, Maynooth, Kildare Ireland; 3 Department of Clinical and Developmental Neuropsychology, Faculty of Behavioral and Social Sciences, University of Groningen, 9712 TS Groningen, The Netherlands

Attention deficit hyperactivity disorder (ADHD) is one of the most common syndromes of childhood. While in the past, ADHD was assumed to be a condition that disappears with maturation and is non-existent in adulthood, it is nowadays understood that ADHD persists into adulthood. Although there is a discussion about the actual percentage of children with ADHD who will still suffer from the disorder in adulthood, there is little doubt that it is a substantial proportion. Data from epidemiologic and follow-up studies indicate a persistence of ADHD across adolescence into adulthood in one to two thirds of diagnosed children with ADHD. The key defining symptoms of ADHD are inattention, hyperactivity and impulsivity, however, motor symptoms of hyperactivity/impulsivity were shown to be less dominant in adult ADHD, whereas attention deficits were found to become more pronounced in adults with ADHD. These core symptoms are pervasive and lead to severe functional impairments of affected adults. Besides these core symptoms, ADHD in adults can produce a wide range of additional cognitive deficits as well as motivational, affective, and social symptoms which can affect nearly every aspect of real-life functioning. In this context, it has to be considered that typical symptoms of ADHD may affect various domains of real-life functioning at the same time. For example, impulsivity of an adult with ADHD may have a negative impact on his or her social relationships, food choice and consumption, risk behaviour, handling of money, etc. Conversely, a specific domain of real-life functioning, such as driving a motor vehicle, may at the same time be adversely affected by various symptoms of ADHD, including impulsivity, inattention, distractibility and lack of flexibility, for instance. High rates of comorbidity further contribute to the impairment in real-life functioning. Real-life functioning is a complex and resource demanding process which requires the integrity of a number of abilities in a dynamic environment with constantly changing requirements. Such abilities include, among others, cognition, self-awareness, coping with stress and flexibility/adaptability. Typical domains of real-life functioning comprise among others social relationships, parenting, diet, social media use, mobility such as driving a motor vehicle, financial planning and decision-making, risk-taking, and vocational functioning.

As real-life functioning is typically impaired in adults with ADHD, this special issue has been put together with the goal of highlighting the significance of real-life functioning of adults with ADHD and the problems they experience. Furthermore, the prerequisites of successful everyday functioning (e.g. neuropsychological functions) and the related concepts (e.g. treatment options and quality of life or circadian rhythm) are considered in the current issue. Various scholars who are experts in their fields contributed to this endeavour by providing their up-to-date applied or fundamental research results. The result is a comprehensive overview of present research activities examining the impact of ADHD on real-life functioning in adults.

Guo and colleagues present data about the neuropsychological functioning of individuals at clinical evaluation of adult ADHD.

Diaz Orueta and colleagues systematically review the literature regarding cognitive functions of adults with ADHD.

Butzbach and colleagues study metacognition in adult ADHD by examining the subjective and objective perspectives on self-awareness of cognitive functioning.

Asadi and colleagues examine unique predictions of general risk-taking behaviour in adult ADHD.

Koerts and colleagues discuss financial judgement determination in adults with ADHD.

Barra and colleagues present a study on the role of stress-coping strategies for life impairments in ADHD.

Godfrey and colleagues examine public perceptions of adult ADHD as indications of stigma.

Young and colleagues review the literature on ADHD and offending.

Fuermaier and colleagues study the nature of work-related problems and impairments of adults with ADHD and explore the association to ADHD symptoms and neuropsychological test performance.

Werling and colleagues investigate screen media use before, during and after the COVID-19 lockdown in ADHD.

Becke and colleagues discuss a new validity index embedded in the Conners’ Adult ADHD Rating Scales and provide information about its development and validation.

Harrison and colleagues perform a comparison of the self-report patterns of analogue versus real world malingerers of ADHD.

Fuermaier and colleagues investigate feigning of ADHD and stimulant misuse among Dutch university students.

Mucci and colleagues perform a naturalistic review of the literature about ADHD treatment across the lifespan.

Young and colleagues review key research in the literature on the assessment and treatment of substance use in adults with ADHD.

Tamburin and colleagues explore whether cognitive dysfunction differs in high-dose benzodiazepine/Z-drug users with and without adult ADHD and the impact of cognitive deficits and adult ADHD on quality of life in this substance-use disorder.

Faltraco and colleagues examine circadian gene expression in ADHD and the influence of norepinephrine, dopamine, atomoxetine and remdesivir.

We hope that this special issue will reflect the wide range of scientific efforts in this exciting field and inspires all those who work in the field of adult ADHD, or plan to do so.

